# A Synthetic Phospholipid Derivative Mediates Ion Transport Across Lipid Bilayers

**DOI:** 10.3389/fchem.2021.667472

**Published:** 2021-04-29

**Authors:** Chenxi Wang, Huiting Yang, Yanxin Xiang, Shihao Pang, Chunyan Bao, Linyong Zhu

**Affiliations:** Shanghai Key Laboratory of Functional Materials Chemistry, School of Chemistry and Molecular Engineering, East China University of Science and Technology, Shanghai, China

**Keywords:** synthetic phospholipids, artificial membrane transporter, channel, relay mechanism, ion selectivity

## Abstract

Inspired by the natural phospholipid structures for cell membrane, a synthetic phospholipid **LC** with an ion recognition group benzo-18-crown-6 (B18C6) moiety was prepared which has been demonstrated to be able to transport ions across the lipid bilayers. Fluorescent vesicle assay shows that **LC** has an excellent transport activity, and the EC_50_ value for K^+^ is 11.2 μM. The voltage clamp measurement exhibits regular square-like current signals with considerably long opening times, which indicates that **LC** achieves efficient ion transport through a channel mechanism and its single channel conductivity is 17 pS. Both of the vesicle assay and patch clamp tests indicate that **LC** has selectivity for Rb^+^, whose ionic radius is larger than the cavity of crown ether. It suggests that the sandwich interaction may play a key role in the ion transport across lipid bilayers. All these results help us to speculate that **LC** transports ions via a channel mechanism with a tetrameric aggregate as the active structure. In addition, **LC** had obvious toxicity to HeLa cells, and the IC_50_ was 100.0 μM after coculture for 36 h. We hope that this simple synthetic phospholipid will offer novel perspectives in the development of more efficient and selective ion transporters.

## Introduction

Cell is the basic unit of structure and function for all organisms. Cell membrane is not only the barrier for cell to survive independently, but also the medium for cell to contact with the surrounding environment and other cells. It can maintain the survival of life through substance exchange, signal transduction and energy conversion. Although some non-polar molecules can be transported inside and outside the cell membrane through free diffusion, the transport of polar solutes and biomacromolecules, such as ions, glucose, amino acids, etc., requires the assistance of specific membrane transporters through carrier or channel mechanisms (Appleman and Lienhard, 1989; Lang et al., 2005; Bröer, 2008; Mosgaard and Heimburg, 2013).

Considering the energy required for the ion to move across the bilayer membrane, the ion transporter should provide enough intermolecular interaction to compensate for the loss of ion hydration energy. Therefore, ion transport through membrane is actually a supramolecular function (Fyles, 2007). Inspired by the functional sophistication of ion transporters in nature, supramolecular chemists have created a variety of synthetic systems to replicate the transport functions by using small molecules and synthetic compounds (Kim and Sessler, 2015; Si et al., 2015; Chen et al., 2018a,b, 2020; Wu et al., 2018; Zhang et al., 2019; Zheng et al., 2020). It is of biological importance to build artificial ion transporters with desired properties, which will not only help to elucidate the possible mechanism by biological transporters, but also to provide early diagnosis and potential medical applications for the treatment of diseases caused by structural and functional abnormalities (such as “channelopathies”) (Zaydman et al., 2012).

In the design of synthetic transporters, most reported compounds have hydrophobic structures to facilitate the insertion of hydrophobic phospholipid bilayer membrane (Kim and Sessler, 2015; Si et al., 2015; Wu et al., 2018; Zheng et al., 2020). However, there are two obvious shortcomings in such a design, including the requirement of organic solvents to solute the compounds and the competitive self-precipitation in physiological solution before inserting in lipid membrane. The use of additional organic solvents increases the biological toxicity and the competitive self-precipitation reduces the transport activity, which affect the further application of these artificial transporters in pharmaceutical areas. Some water-soluble peptides have been reported to undergo supramolecular self-assembly to transport ions when exposed to membranes (Shank et al., 2006; Elliott et al., 2008). Complex design and synthetic procedures limit the structural diversity of water-soluble peptide transporters to a certain extent. It is known that the main component of cell membrane is the mixture of phospholipids, and the membrane is composed of two layers of phospholipid molecules arranged symmetrically. If the structure of phospholipids is combined with an ion recognition group, it provides an alternative option to develop efficient ion transporters. This kind of ion transporters will have the following two advantages: the zwitterionic structure can solve the above water solubility problem, and the phospholipid-like structure also increases its interaction with the membrane. Up to now, synthetic phospholipids with ion transport ability have very seldom been reported. A typical work was reported by the Smith group, in which a series of phosphatidylcholine derivatives containing different Cl^−^ recognition groups have been confirmed to transport Cl^−^ across the lipid bilayers by a relay mechanism (McNally et al., 2008). They detailed studied the effect of membrane thickness on Cl^−^ influx rates and proposed two possible relay mechanisms by a dimeric or a tetrameric aggregate.

**Scheme 1:**
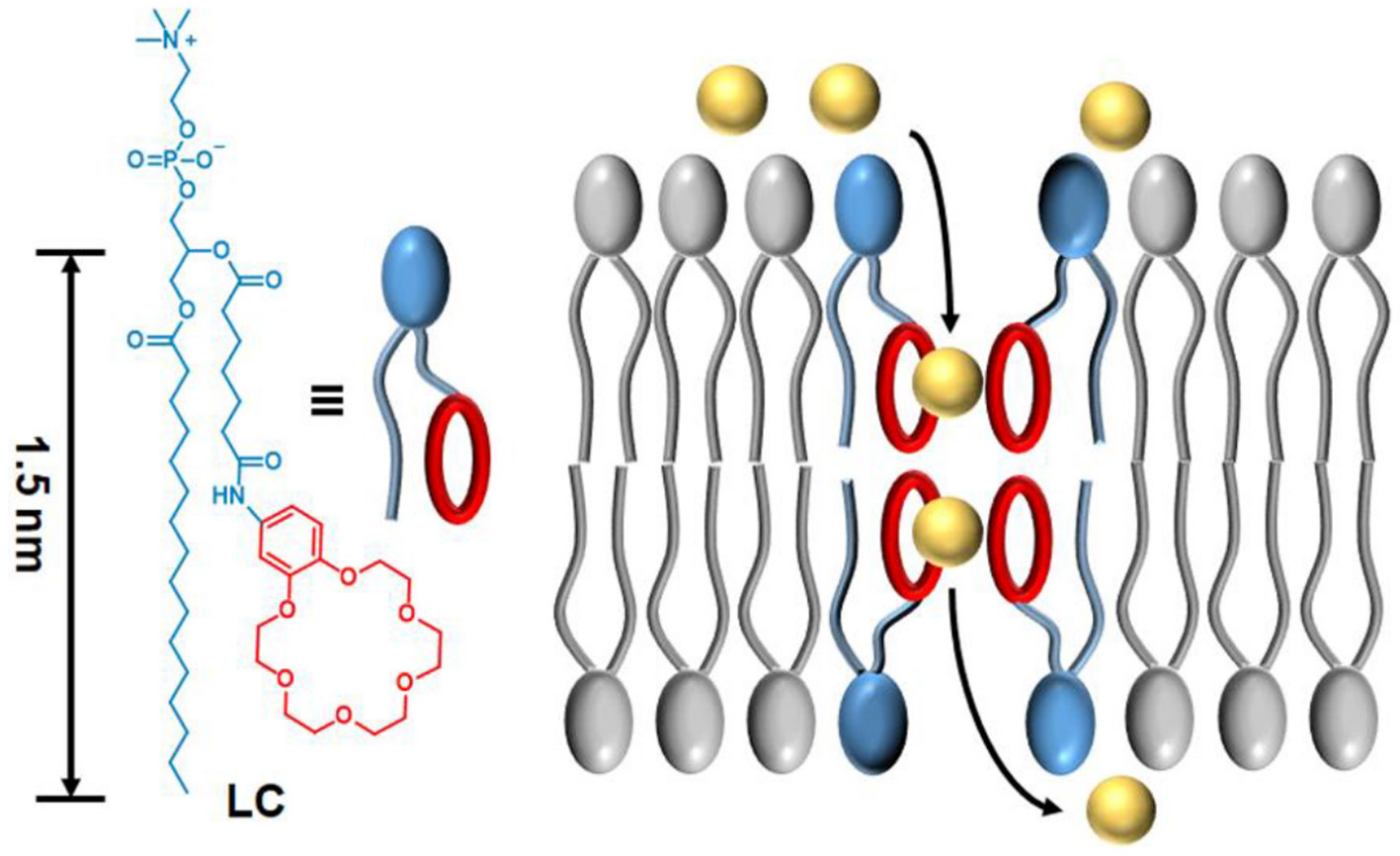
The molecular structure of synthetic phospholipid **LC** and the proposed transport mechanism across a lipid membrane.

Taking inspiration from above relay mechanism, we herein reported a new synthetic phospholipid **LC** which can perform cation transport across the lipid membranes. As shown in Scheme 1, transporter **LC** contains a benzo-18-crown-6 (B18C6) group with relatively high affinity for cations. The amphipathic of **LC** allows all transport analysis to be performed in buffer solutions without using any organic solvents, and the phospholipid structure enables **LC** to strongly interact with the membrane, thus achieving efficient ion transport from one side of the membrane to the other. Ion selectivity analysis and voltage clamp single-channel current recording suggested that ion transport of **LC** is more likely to be mediated by the channel mechanism of a tetrameric aggregate. Excellent transport activity and cytotoxicity to cancer cells will facilitate the application of synthetic phospholipid transporters in biomedical research.

## Materials and Methods

### Materials

All starting materials were obtained from commercial suppliers and were used without further purification unless otherwise stated. All air- or moisture-sensitive reactions were performed using oven-dried or flame-dried glassware under an inert atmosphere of dry argon. Egg yolk phosphatidylcholine (EYPC) and 1,2-diphytanoyl-*sn*-glycero-3-phosphocholine (DPhPC) were obtained from Avanti Polar lipids as powder. 8-hydroxy-1,3,6-pyrenetrisulfonate (HPTS) and Triton X-100 were obtained from Sigma Aldrich and used without further purification.

### Characterizations

Proton and carbon nuclear magnetic resonance spectra (^1^H, ^13^C NMR) were recorded on a Bruker Avance 400 MHz/600 MHz spectrometer. Mass spectra were recorded on a Micromass GCTTM and a Micromass LCTTM. Fluorescence measurements were performed on a Varian Cary Eclipses fluorescence spectrometer equipped with a stirrer and a temperature controller (kept at 25°C unless otherwise noted). A Mini-Extruder used for the preparation of LUVs was purchased from Avanti Polar lipids. The size of EYPC vesicles was determined using a Delsa™ Nano Submicron Particle Size and Zeta Potential Particle Analyzer (Beckman Coulter Inc., USA). Preparative reverse phase HPLC was performed using a Waters 2454 Multisolvent Delivery System with a Waters 2489 UV/visible detector operating at 254 nm.

### Synthesis of Compounds

#### Compound S1

To a solution of 4-nitrocatechol (1.0 g, 6.45 mmol) in acetonitrile (300 mL) was added **S0** (Chen et al., 2018a) (8.8 g, 16.1 mmol) and K_2_CO_3_ (5.5 g, 40 mmol). The mixture was refluxed at 90°C for 12 h. Then, the mixture was cooled to room temperature and filtered. Finally, the solvent was removed under vacuum to obtain the crude product, which was further purified by silica gel flash column chromatography (DCM/MeOH = 100:1) to afford yellow powder compound **S1** (1.66 g, 72% yield). ^1^H NMR (600 MHz, CDCl_3_) δ = 7.89 (dd, *J* = 8.9, 2.6 Hz, 1H), 7.74 (d, *J* = 2.7 Hz, 1H), 6.89 (d, *J* = 8.9 Hz, 1H), 4.29–4.19 (m, 4H), 4.01–3.91 (m, 4H), 3.78 (dq, *J* = 5.9, 2.2 Hz, 4H), 3.74–3.70 (m, 4H), 3.69 (s, 4H). ^13^C NMR (151 MHz, CDCl_3_) δ = 154.46, 148.50, 141.43, 117.97, 111.34, 108.30, 71.04, 70.64, 69.20. MS (ESI): m/z: Calcd. for C_16_H_24_${\text{NO}}_{8}^{+}$ [M+H]^+^: 358.2. Found: 358.1.

#### Compound S2

To a solution of **S1** (5.0 g, 14 mmol) in dichloromethane (200 mL) was added 10% Pd/C (0.2 g). The mixture was stirred at room temperature under hydrogen (balloon) atmosphere and monitored by thin layer chromatography. After the reduction reaction was completed, Pd/C was filtered and the solvent was removed under vacuum. The crude product was further purified by silica gel flash column chromatography (DCM/MeOH = 100:10) to afford colorless oil compound **S2** (3.4 g, 74% yield). ^1^H NMR (400 MHz, CDCl_3_) δ = 6.72 (d, *J* = 8.4 Hz, 1H), 6.30 (d, *J* = 2.6 Hz, 1H), 6.23 (dd, *J* = 8.4, 2.6 Hz, 1H), 4.12–4.04 (m, 4H), 3.91 (dd, *J* = 5.7, 3.7 Hz, 2H), 3.87 (dd, *J* = 5.8, 3.8 Hz, 2H), 3.78–3.69 (m, 8H), 3.68 (s, 4H). ^13^C NMR (151 MHz, CDCl_3_) δ = 150.00, 142.28, 116.77, 108.19, 103.31, 70.75, 69.93, 69.66, 68.70. MS (ESI): m/z: Calcd. for C_16_H_26_${\text{NO}}_{6}^{+}$ [M+H]^+^: 328.2. Found: 328.2.

#### Compound **S3**

Under Argon atmosphere, 1-chloro-N,N-2-trimethylprop-1-en-1-ylamine (2.7 g, 20 mmol) was added to a solution of monoethyl heptanedioate (3.0 g, 15.9 mmol) in dry dichloromethane (50 mL). The mixture was stirred at room temperature for 3 h. After removing the solvent under vacuum, the obtained acyl chloride was re-dissolved in dry dichloromethane (20 mL) for use. Then, to a solution of **S2** (3.3 g, 10 mmol) in 20 mL dichloromethane was added above acyl chloride solution dropwise. The obtained mixture was kept stirring at room temperature for 12 h and the crude product was obtained under vacuum, which was further purified by silica gel flash column chromatography (DCM/MeOH = 100:5) to afford white powder compound **S3** (3.1 g, 62% yield). ^1^H NMR (400 MHz, CDCl_3_) δ = 7.54 (d, *J* = 9.2 Hz, 1H), 7.37 (d, *J* = 2.4 Hz, 1H), 6.87 (dd, *J* = 8.6, 2.4 Hz, 1H), 6.79 (dd, *J* = 8.6, 1.7 Hz, 1H), 4.21–4.05 (m, 6H), 3.89 (dt, *J* = 6.7, 3.2 Hz, 4H), 3.81–3.65 (m, 12H), 2.32 (dtd, *J* = 11.9, 7.5, 4.3 Hz, 4H), 1.68 (ddt, *J* = 34.4, 15.2, 7.5 Hz, 4H), 1.47–1.33 (m, 2H), 1.25 (td, *J* = 7.1, 3.1 Hz, 3H). ^13^C NMR (151 MHz, CDCl_3_) δ = 173.80, 171.76, 145.70, 142.23, 133.50, 112.56, 110.73, 104.42, 60.28, 50.83, 45.89, 34.10, 28.72, 24.66, 14.27, 8.65. MS (ESI): m/z: Calcd. for C_25_H_39_NO_9_K^+^ [M+K]^+^: 536.2. Found: 536.3.

#### Compound S4

To a solution of **S3** (1.0 g, 2 mmol) in MeOH/H_2_O (V:V = 9:1, 100 mL) was added KOH (0.5 g, 8.9 mmol). The mixture was stirred at room temperature for 12 h. Then, the pH of the solution was adjusted to 7.0 by addition of HCl aqueous solution (1 M). The solvent was removed under vacuum and the residue was dissolved with 20 mL MeOH. After filtratio, the residue was purified by reverse phase preparative HPLC (MeOH/H_2_O = from 30:70 to 100:0 in 25 min) to afford white powder compound **S4** (0.4 g, 42.6% yield). ^1^H NMR (400 MHz, CDCl_3_) δ = 8.97 (s, 1H), 7.37 (d, *J* = 2.3 Hz, 1H), 7.09 (dd, *J* = 8.7, 2.2 Hz, 1H), 6.70 (d, *J* = 8.7 Hz, 1H), 4.06 (t, *J* = 4.7 Hz, 4H), 3.83 (dd, *J* = 16.3, 4.3 Hz, 4H), 3.74–3.58 (m, 12H), 2.27 (dt, *J* = 41.0, 7.4 Hz, 4H), 1.56 (dp, *J* = 46.2, 7.2 Hz, 4H), 1.27 (d, *J* = 9.0 Hz, 2H). ^13^C NMR (151 MHz, CDCl_3_) δ = 176.56, 170.68, 147.90, 144.25, 131.43, 113.59, 111.53, 106.09, 69.66, 68.52, 67.72, 52.42, 36.04, 32.70, 28.68, 27.43, 24.17, 23.30. MS (ESI): m/z: Calcd. for C_23_H_35_NO_9_Na^+^ [M+Na]^+^: 492.2. Found 492.1.

#### Compound **LC**

To a solution of 1-palmitoyl-2-hydroxy-sn-glycero-3-phosphocholine (**Lyso PC**, 20.0 mg, 0.04 mmol) in DMF (1 mL) was added **S4** (94.0 mg, 0.2 mmol), EDC (38.0 g, 0.2 mmol) and DMAP (5.0 mg, 0.04 mmol). The mixture was stirred at room temperature for 2 days. After the reaction was completed, the mixture was diluted with 20 mL MeOH/H_2_O (V:V = 1:1) and purified by reverse phase preparative HPLC (MeOH/H_2_O = from 50:50 to 100:0 in 25 min) to afford colorless oil compound **LC** (13 mg, 34.3% yield). ^1^H NMR (400 MHz, CDCl_3_) δ = 10.06 (s, 1H), 7.51 (d, *J* = 2.3 Hz, 1H), 7.17 (dd, *J* = 8.6, 2.2 Hz, 1H), 6.77 (d, *J* = 8.7 Hz, 1H), 5.24 (tt, *J* = 7.2, 3.8 Hz, 1H), 4.22–4.00 (m, 8H), 3.85 (q, *J* = 4.1 Hz, 4H), 3.69 (t, *J* = 4.7 Hz, 4H), 3.64 (d, *J* = 9.9 Hz, 8H), 3.53 (d, *J* = 4.9 Hz, 2H), 3.14 (s, 9H), 2.51–2.17 (m, 6H), 1.62 (ddq, *J* = 37.2, 14.3, 7.1 Hz, 6H), 1.40 (q, *J* = 7.1 Hz, 2H), 1.25 (s, 26H), 0.88 (t, *J* = 6.6 Hz, 3H). ^13^C NMR (151 MHz, CDCl_3_) δ = 173.60, 173.36, 172.54, 148.23, 144.26, 133.82, 113.74, 112.66, 106.83, 70.82, 70.37, 70.22, 69.57, 69.49, 68.91, 68.46, 66.44, 64.11, 62.73, 59.06, 54.44, 36.93, 34.12, 31.93, 29.71, 29.37, 29.16, 28.06, 24.58, 22.70, 14.14. HRMS (ESI): m/z: Calcd. for C_47_H_84_N_2_O_15_PNa^2+^ [M+Na+H]^2+^: 485.2748. Found 485.2738.

### Dynamic Light Scattering Analyses

(a) Preparation of large unilamella vesicles (LUVs): To an EYPC solution (10.0 mg, 400 μL, 25.0 mg mL^−1^ in CHCl_3_) was added cholesterol (1.0 mg, 100 μL, 10 mg mL^−1^ in CHCl_3_). The solvent was evaporated by a slow stream of nitrogen, followed by drying under vacuum for 12 h. Then, the lipid membrane was hydrated with 500 μL HEPES buffer (10 mM HEPES, 100 mM KCl, pH = 7.0) for 2 h at 37°C. The obtained suspension was subjected to seven freeze–thaw cycles and extruded 21 times through a 100 nm polycarbonate membrane. Finally, the large unilamella vesicles (LUVs) was purified with size exclusion column chromatography (SephadexG-25) and further diluted to 12.9 mL with HEPES buffer to afford a stock solution with a lipid concentration of 1.0 mM (assuming 100% of lipids were incorporated into liposomes).

(b) Preparation of LC per-incorporated LUVs: LC (0.25 mg, 100 μL, 2.5 mg mL^−1^ in CHCl_3_, 10 mol% to EYPC) was added to a mixture containing EYPC solution (2.0 mg, 80 μL, 25 mg mL^−1^ in CHCl_3_) and cholesterol (0.2 mg, 20 μL, 10 mg mL^−1^ in CHCl_3_). The solvent was then evaporated by a slow stream of nitrogen, followed by drying under vacuum for 12 h. The obtained lipid membrane was hydrated with 100 μL HEPES buffer (10 mM HEPES, 100 mM KCl, pH = 7.0) in a shaker for 2 h (37°C, 180 rad/min). The suspension was subjected to seven freeze–thaw cycles and extruded 21 times through a 100 nm polycarbonate membrane. Finally, the mixture was purified with size exclusion column chromatography (SephadexG-25) and further diluted to 2.58 mL with HEPES buffer to afford a stock solution with a lipid concentration of 1.0 mM (assuming 100% of lipids were incorporated into liposomes).

Four samples were characterized by DLS. In a typical experiment, 2900 μL of HEPES buffer (10 mM HEPES, 100 mM KCl, pH = 7.0) was transferred to a quartz cuvette followed by the addition of 100 μL of above stocked liposome solutions. “LUVs” means the sample prepared by adding (a) stocked liposome solution; “LUVs+LC” means the sample prepared by adding (a) stocked liposome solution and LC (10 μL stock solution in H_2_O, 10.0 μM final concentration); “LC per-incorporated LUVs” means the sample prepared by adding (b) stocked liposome solution; and “LC + Triton X-100” means the sample prepared by adding (a) stocked liposome solution and Triton X-100 (10 μL stock solution in H_2_O, 10.0 μM final concentration).

### Transmission Electron Microscope Analyse

Preparation of the TEM sample: **LC** was dissolved in H_2_O (10.0 μM) and incubated in a shaker for 20 min (37°C, 180 rad/min). Then the mixture was dropped in support films and negatively stained by uranyl acetate.

### Analyses of Transport Activity of LC

Preparation of LUVs⊃HPTS: The procedure was similar as for LUVs except a HEPES containing 8-hydroxypyrene-1,3,6-trisulfonic acid (HPTS, 1.0 mM) was used for the hydration process.

LUVs⊃HPTS assay: In a typical experiment, 2900 μL of HEPES buffer (10 mM HEPES, 100 mM KCl, pH = 7.0) was transferred to a quartz cuvette followed by addition of 100 μL above LUVs⊃HPTS solution. The cuvette was placed in the fluorescence instrument with slow stirring condition by a magnetic stirrer equipped in the instrument (at *t* = 0 s). The time-dependent change in fluorescence intensity (λ_em_ = 510 nm) was monitored at two excitation wavelengths simultaneously (I_450_ : λ_ex_ = 450 nm, I_405_ : λ_ex_ = 405 nm), during the addition of base (30 μL, 0.5 M KOH, ΔpH = 0.8) at *t* = 50 s, **LC** (10 μL stock solution in H_2_O, 0–50.0 μM final concentration) at *t* = 100 s, and 60 μL of 5% Triton X-100 aqueous solution at *t* = 550 s. All the temperature was kept at 25°C by a stirrer and a temperature controller. Time courses of fluorescence intensity *I*_*F*_ were obtained by ratiometric analysis (*R* = I_450_/I_405_) and normalization according to Equation 1,

(1) $$\begin{array}{l} I_{F}\;=\;\left(R\;-\;R_{100}\right)/\left(R_{\infty }\;-\;R_{100}\right)\; \end{array}$$

where *R*_100_ is *R* before addition of transporter and *R*_∞_ is *R* after addition of Triton X-100. The solvent H_2_O (10 μL) was also monitored as the fluorescence background. Finally, *I*_*t*_ at 550 s just before the addition of Triton X-100 was defined as transmembrane activity *Y* (*Y* = *I*_*F*_–*I*_H2O_), which was further analyzed with Hill Equation 2 to give effective concentration EC_50_ and the Hill coefficient n,

(2) $$\begin{array}{l} Y\;=\;Y_{\infty }\;+\;\left(Y_{0}\;-\;Y_{\infty }\right)/\left(1\;+\;{\left(\frac{c}{{EC}_{50}}\right)}^{n}\right) \end{array}$$

Where *Y*_0_ is *Y* in absence of transporter (normally defined as 0), *Y*_∞_ is *Y* with excess transporter (normally defined as 1) and *c* is the transporter concentration.

For clarity, the data before the addition of transporter was deleted and time (*X*-axis) was changed to start from the point of transporter addition (i.e., *t* = 100 s was normalized to *t* = 0 s) to the end point of experiment (i.e., *t* = 550 s was normalized to *t* = 450 s).

Ion selectivity: 100 μL LUVs⊃HPTS solution was added to 2900 μL gently stirred, thermostatic buffer (10 mM HEPES, 100 mM M^+^Cl^−^, pH 7.0, where M^+^ = Li^+^, Na^+^, K^+^, Rb^+^, Cs^+^) in a fluorimetric cell. The time dependent change in fluorescence intensity was monitored and analyzed as described above (final concentration of transporter is 10.0 μM) to obtain the fractional transmembrane activity.

FCCP assay: The vesicles preparation, experimental procedure and data processing were same as above LUVs⊃HPTS assay except a solution containing carbonyl cyanide-4-(trifluoromethoxy)-phenylhydrazone (FCCP, 10 μL in HEPES, 5.0 μM) was added at *t* = 75 s. Similarly, the initial 100 s data was removed after normalization. Data analysis and comparison was in the same way as stated above.

### Patch Clamp Measurements

DPhPC lipid in chloroform was dried under a stream of nitrogen and then under vacuum each for 4 h. The obtained membrane was re-dispersed in decane with a concentration at 20.0 mg mL^−1^. The solution was used to precoat a 200 μm hole of a polystyrene cup held by a chamber upon which a planar lipid bilayer membrane was formed. The cup (*cis*, ground) and chamber (*trans*) were filled with 1 mL 1.0 M KCl solution. Formation of membrane was monitored by measuring membrane capacitance. **LC** (10 μL stock solution in H_2_O, 5.0 μM final concentration) was added to the *cis* side of the chamber and the solution was stirred for 2 min. Various holding potentials were applied and the channel responses were recorded. Ag/AgCl electrodes were used to impose voltages and record currents across the membrane. The patch clamp workstation (Warner Instruments) was used for all experiments. The currents were measured by a Warner BC-535 bilayer clamp amplifier and collected using the Digidata 1550A data acquisition system. All data was filtered with 8-pole Bessel filter.

The selectivity calculation of K^+^/Rb^+^: The cup (*cis*, ground) was filled with 1 mL 1.0 M RbCl solution and the chamber (*trans*) was filled with 1 mL 1.0 M KCl solution. The other conditions are the same as above. The selectivity of **LC** for K^+^ over Rb^+^, defined as the permeability ratio of two ions, was calculated by using the simplified Goldman-Hodgkin-Katz (GHK) equation (Chui and Fyles, 2012; Chen et al., 2018a):

(3) $$\begin{array}{l} \frac{P_{K^{+}}}{P_{{Rb}^{+}}}\;=\;e^{\frac{-\;\varepsilon_{rev}F}{RT}} \end{array}$$

where **ε**_**rev**_ is the reversal potential (the potential of zero current); *R* is the universal gas constant (8.314 J K^−1^ mol^−1^); *T* the temperature in Kelvin (300 K); *F* is the Faraday's constant (96485 C mol^−1^); *P* is the permeability of **LC** for ions.

### *In vitro* Cytotoxicity Assay

CCK-8 assay was used to evaluate *in vitro* cytotoxicity of HeLa cells. HeLa cells were first cultured in Dulbecco's modified Eagle medium (DMEM) supplemented with 10% fetal bovine serum (FBS), penicillin (100 units mL^−1^), and streptomycin (100 mg mL^−1^) at 37°C in a 5% CO_2_ incubator for 3 days. For cytotoxicity assay, HeLa cells were seeded in a 96-well plate at an initial density of 5,000 cells per well in 200 μL of complete DMEM medium. After incubating for 24 h, DMEM was replaced with fresh medium, and the cells were treated with samples at varying concentrations. After the coculture for 12, 24, and 36 h, respectively, the medium in each well was removed and replaced by 100 μL of fresh DMEM. Then, CCK-8 reagent (10 μL) was added to each well and the cells were further incubated for 3 h at 37°C. Finally, the absorbance at 450 nm was recorded by a microplate reader, and the data were averaged from at least six trials.

## Results and Discussion

**Figure 1:**
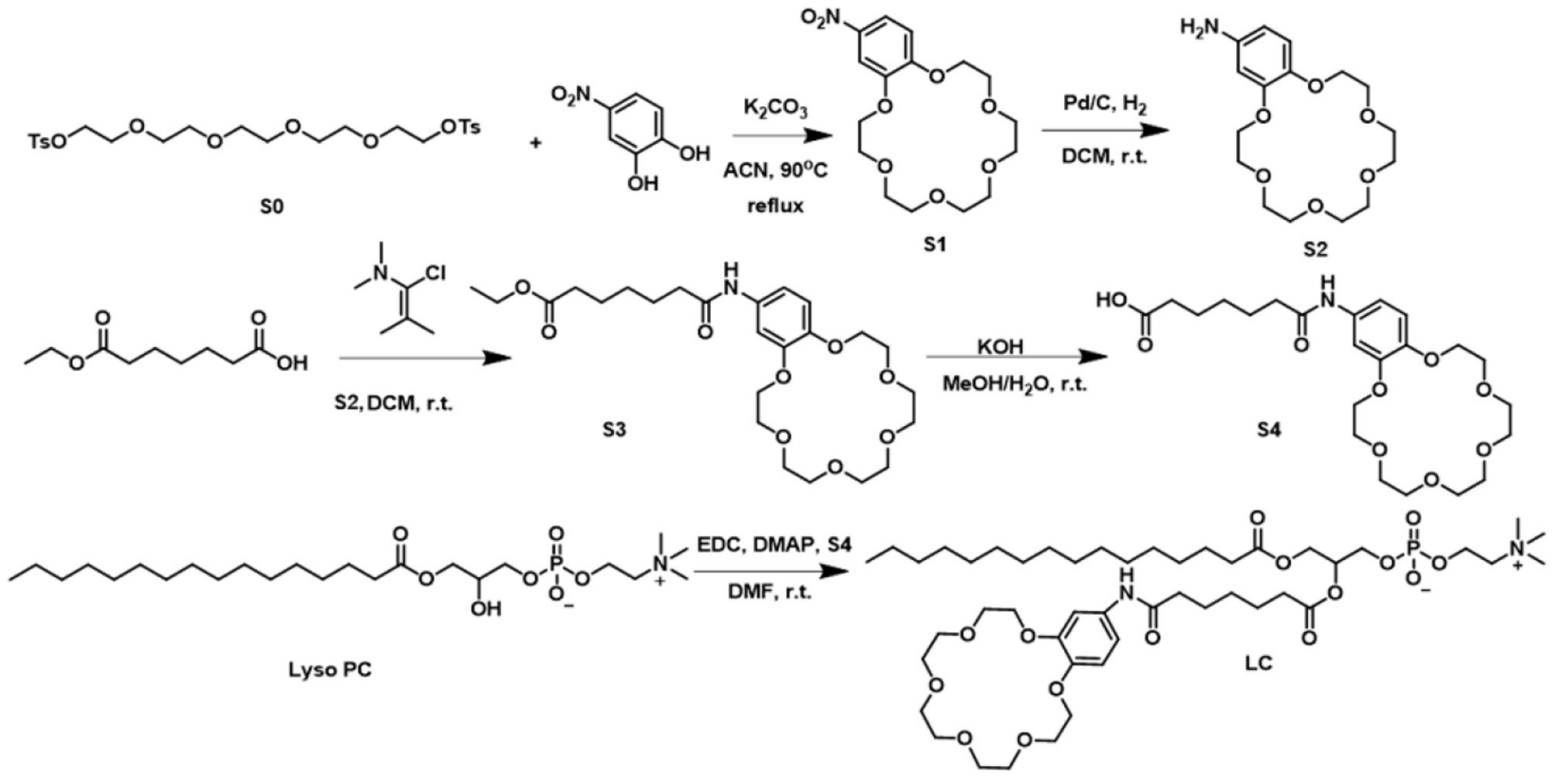
Synthesis procedure of **LC**.

**Figure 2:**
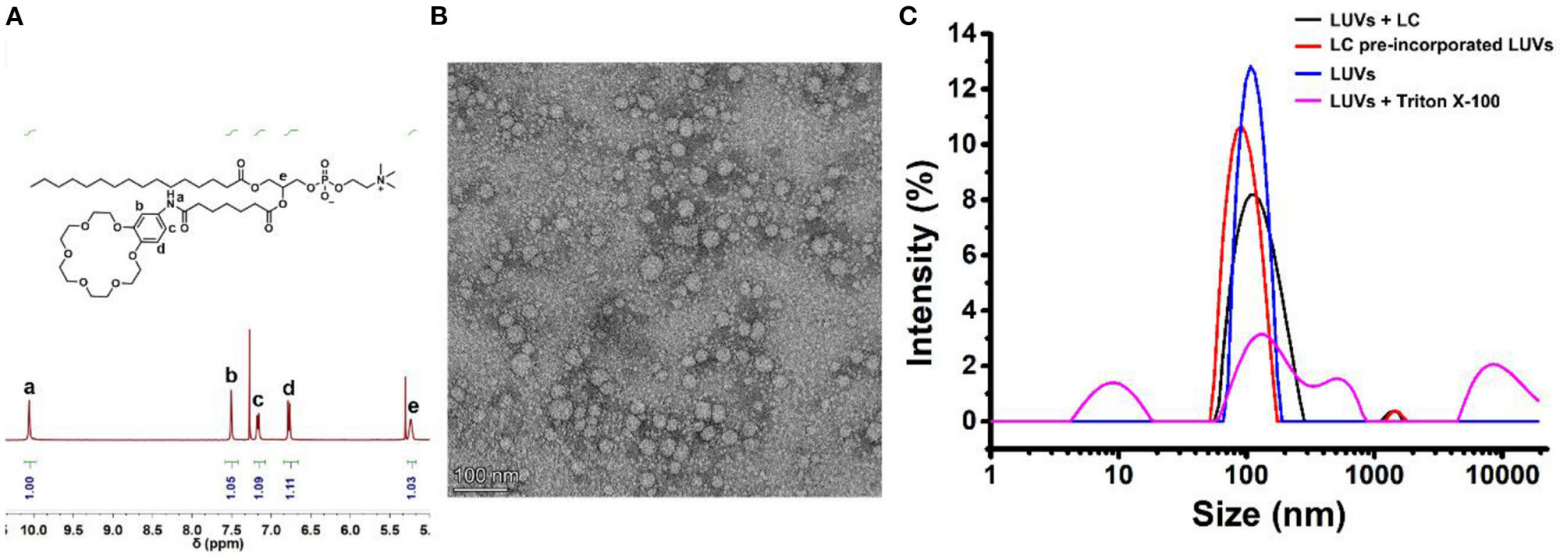
**(A)** The part ^1^H NMR spectra for purified **LC** by reverse phase preparative HPLC. **(B)** TEM image of self-assemble of LC in water, the scale bar is 100 nm. **(C)** DLS analysis of the LUVs. “+LC” means **LC** (10.0 μM) was added directly into LUVs; “LUVs-LC” means **LC** per-incorporated LUVs as prepared; “+Triton X-100” means the LUVs with the addition of Triton X-100 (10.0 μM); and “Blank LUVs” means the LUVs as prepared.

Phospholipid derivative **LC** was synthesized by the following five steps: (1) cyclization of crown ether; (2) reduction of nitro group; (3) coupling of amide; (4) hydrolysis of ester; and (5) condensation of ester (Figure 1). All the compounds were confirmed by both NMR and mass spectra. Although the synthetic procedure is simple, the final product **LC** cannot be purified by conventional strategies due to its zwitterionic structure. Finally, **LC** was purified by reverse phase preparative HPLC. The appearance of characteristic shifts of H_a_, H_b_, H_c_, and H_d_ assigned to B18C6 confirmed the successful incorporation of an ion recognition group into a nature lipid **Lyso PC** (1-palmitoyl-2-hydroxy-sn-glycero-3-phosphocholine) (Figure 2A). **LC** shows excellent water dispersibility due to its amphiphilic character, and it can self-assembled into vesicles with a diameter of 20–30 nm (Figure 2B), which facilitates all the characterizations under physiological conditions. According to the CPK model, the molecular length of **LC** is about 2.3 nm, which is comparable to the length of typical phospholipids (such as EYPC) used in biomimetic membrane. The similar molecular structure and matching molecular length make it possible for **LC** to associate and insert in the lipid membranes via membrane fusion (Kurihara et al., 2011, 2015).

Then, the influence of **LC** on the integrity of the biomimetic membrane was detected by DLS method with large unilamellar lipid vesicles (LUVs) as samples. As shown in Figure 2C, the particle size distribution was only slightly larger than that of the blank LUVs after the addition of **LC**. However, the addition of surfactant Triton X-100 completely decomposed LUVs. It suggests that the addition of **LC** does not destroy the integrity of the membrane. We also tried to prepare LUVs by pre-incorporating **LC** with the EYPC lipids. The comparable vesicle size and narrow particle size distribution further confirmed above suggestion, i.e., **LC** can associate with LUVs without damaging the lipid membranes. This provides a premise for **LC** to transport ions across the membrane like natural transporters.

**Figure 3:**
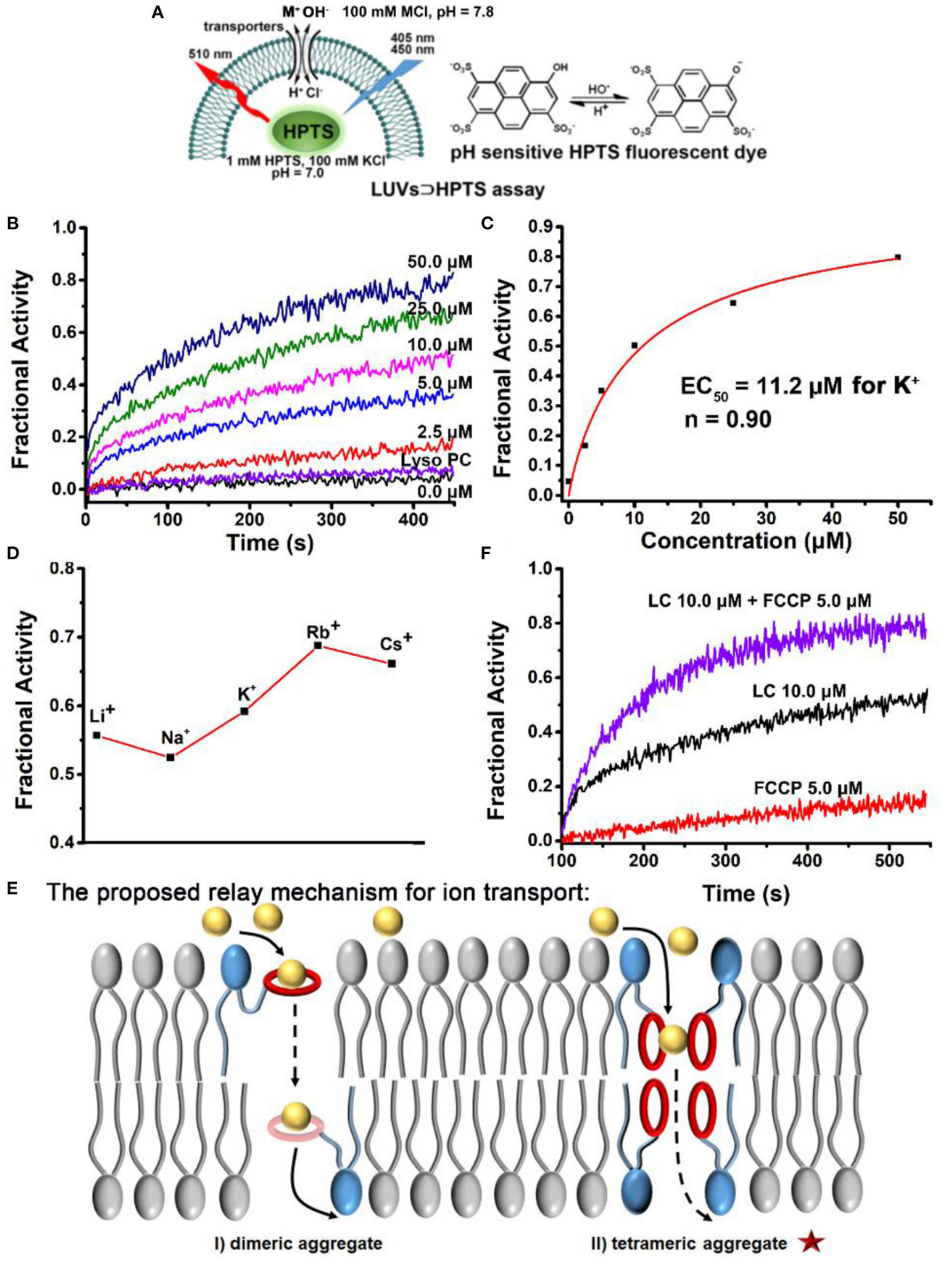
**(A)** Schematic illustration of the pH-sensitive LUVs⊃HPTS assay. **(B)** Ion transport activities of **LC** in different concentrations (0.0~50.0 μM, final concentrations). The activity of **Lyso PC** at 50.0 μM is compared. **(C)** Concentration-activity curves for K^+^ transport. The red lines are the fitted curves from Hill equation (Equation 2) and EC_50_ is 11.2 μM for K^+^. **(D)** Ion selectivity LUVs⊃HPTS assay of **LC** at 10.0 μM. **(E)** Schematic representation of the proposed mechanism for ion transport of **LC** across a lipid membrane. **(F)** FCCP assay, the comparison of ion transport activity of **LC** (10.0 μM) in the absence and presence of FCCP at 5.0 μM, respectively. The activity of FCCP was observed and compared.

Next, to investigate the transmembrane active of **LC**, a well-established fluorescence assay with LUVs was carried out, in which a pH-sensitive fluorescent probe, 8-hydroxypyrene-1,3,6-trisulfonic acid (LUVs⊃HPTS) was entrapped. A pH-gradient (ΔpH = 0.8) was applied by addition of KOH solution in the extravesicular buffer, then the addition of transporters induced the pH gradient collapse via a H^+^ efflux or OH^−^ influx. By monitoring the change in the ratio of fluorescence intensity at 510 nm (I_450_/I_405_) of HPTS (Figure 3A), the ion transport efficiency can be reflected, in which fractional activity was used and compared. As illustrated in Figures 3B,C, a detailed examination of the ion transport of **LC** was carried out by varying their concentrations. Compared with **Lyso PC**, **LC** exhibited much higher transmembrane activity for K^+^ with an EC_50_ value of 11.2 μM (the concentration of transporter required to achieve 50% activity). To explore the B18C6 action on the ion transport of **LC**, cation selectivity were recorded and determined by varying extravesicular cations. As illustrated in Figure 3D, variation of external cations (M^+^ = Li^+^, Na^+^, K^+^, Rb^+^, and Cs^+^) provided different transmembrane activity and followed the order Rb^+^≥Cs^+^>K^+^>Li^+^≥Na^+^. It is quite different to previous reported B18C6-contained transporters (Chen et al., 2018a; Li et al., 2020), which exhibited K^+^ selectivity due to the higher affinity. Compared with K^+^ transport, **LC** showed a slightly active toward Li^+^ and Na^+^ with smaller sizes, and a highly active toward Rb^+^ and Cs^+^ with bigger sizes. The ionic radii of Rb^+^ (1.52 Å) and Cs^+^ (1.67 Å) are too large to fit into the cavity of 18-membered ring crown ethers with a radius of 1.3–1.5 Å (Kikuchi and Sakamoto, 2000). Therefore, it is considered that a second crown ether molecule participates ion recognition and a sandwich coordination of B18C6 moieties toward Rb^+^ and Cs^+^ may play a key role for ion transport across the lipid bilayers (Kim et al., 1999; Lamoureux and Roux, 2006; Sun et al., 2015; Schneider et al., 2017). Based on the mechanism suggested by Smith et al. (McNally et al., 2008), that is, the relay mechanism by a dimeric or a tetrameric aggregates (Figure 3E), the ion selectivity shown by **LC** indicates that its transport mechanism is more inclined to the latter. In order to determine whether the ion transport is via a H^+^/M^+^ antiport or a M^+^/OH^−^ symport mechanism, the transmembrane activity of **LC** in the presence of carbonyl cyanide-4-(trifluoromethoxy)-phenylhydrazone (FCCP, a H^+^ selective transporter) were studied by LUVs⊃HPTS assay. As illustrated in Figure 3F, the addition of FCCP caused obviously increased activity (from 50 to 80% by adding 5.0 μM FCCP), in contrast, the same amount of FCCP itself resulted <10% activity. This result support that M^+^/OH^−^ symport mechanism balanced the charges inside and outside of the LUVs (Saha et al., 2016; Chen et al., 2018b, 2021).

**Figure 4:**
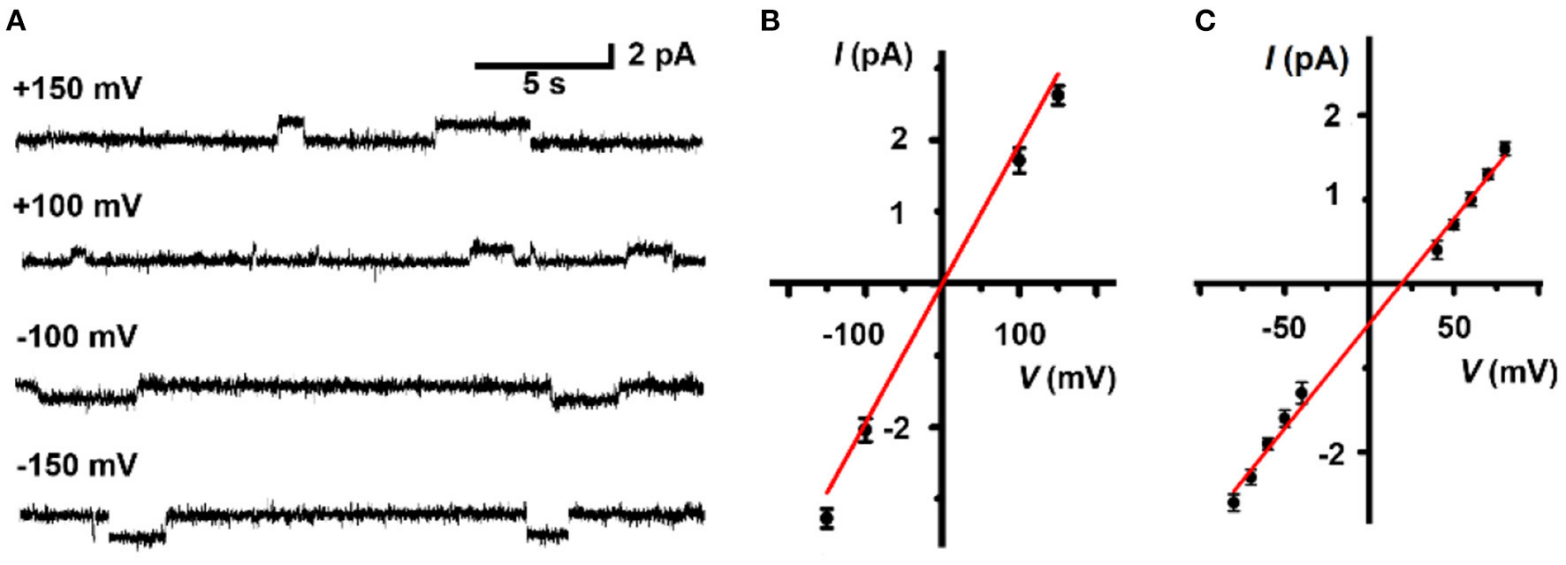
**(A)** Representative current recording and **(B)** linear I-V plots of **LC** (5.0 μM) at various holding potentials in a symmetrical 1 M KCl solution. **(C)** Linear I-V plots of **LC** (5.0 μM) in an asymmetrical solution. 1.0 M KCl in *trans* chamber and 1.0 M RbCl in *cis* chamber. The reversal potential (the potential of zero current) is 18.9 mV.

Finally, to further scrutinize the transport mechanism of **LC**, a patch-clamp technique on planar lipid bilayer membranes (BLMs) was employed to record single-channel current traces. The addition of **LC** led to conductance traces at various holding potentials and the representative signals are shown in Figure 4A. The appearance of regular square-like signals with considerably long opening times suggests that **LC** transports ions as a stable channel or pore across the bilayer membrane. The signals scales linearly in current–voltage relationships in the range of −150 to +150 mV (Figure 4B), and the ohmic I–V profiles allows the evaluation of a single channel conductivity of 17 pS, suggesting effective ion transport across the BLMs. We further performed a patch-clamp assay to quantitatively compare the permeability ratios of these ions by determining reversal potentials (Figure 4C), which revealed a K^+^/Rb^+^ permeability ratio (${\text{P}}_{\text{K}}^{+}$/${\text{P}}_{\text{Rb}}^{+}$) of 0.48 by the calculation of Goldman-Hodgkin-Katz (GHK) equation. Combined with the results of ion selectivity in LUVs⊃HPTS assay, the channel-like current signal further inspired us to speculate that **LC** transports ion through a channel mechanism of a tetrameric aggregate as proposed in Scheme 1.

**Figure 5:**
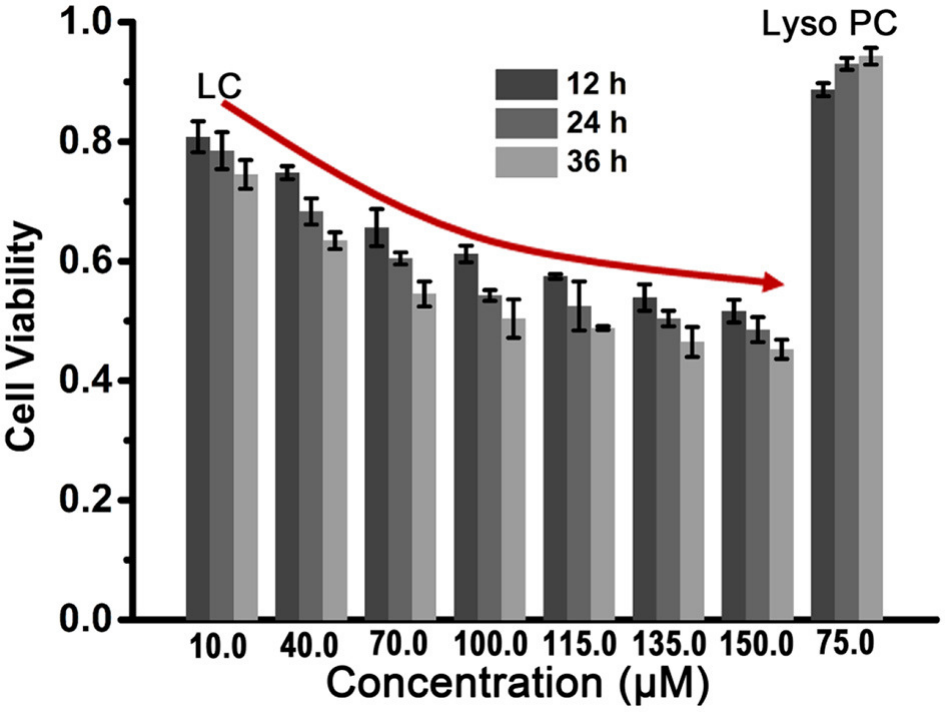
The cytotoxicity analysis of **LC** to HeLa cells determined by CCK-8 assay. **Lyso PC** at 75.0 μM was used as a contrast.

Based on the amphipathic characteristic and the efficient ability for ion transport of **LC**, we expect that **LC** can exhibit potent anticancer ability by changing membrane permeability and disrupting ion homeostasis (Gokel and Negin, 2013). In order to verify above expectation, a series of **LC** cytotoxicity experiments were carried out and HeLa cells were selected as the cell model. After fed HeLa cells with different concentrations of **LC** for 12, 24, and 36 h, respectively, the cell viability was determined by CCK-8 assay and expressed by the ratio to the control group without any samples. As illustrated in Figure 5, **LC** displays considerable toxicity toward HeLa cells, and the cell viability exhibits a dose-response, that is, the cytotoxicity increased with the increase of concentration (IC_50_ ≈ 100.0 μM for 36 h co-culture). However, **Lyso PC** shows good cytocompatibility even when the concentration increases to 75.0 μM (maximum soluble concentration), and the cell viability is higher than 88%. These preliminary results show that **LC** has a potential application as an anticancer drug, although its cytotoxicity is lower than that of classical anticancer drugs, such as doxorubicin hydrochloride with IC_50_ value of 4.3 μM under the same conditions (Zhou et al., 2020).

## Conclusion

In conclusion, we designed and synthesized a water-soluble phospholipid **LC** with an ion recognition group B18C6 for cation transport across the lipid membranes. Both vesicle and planar lipid bilayer experiments verify the efficient ion transport; the change of cation species and the addition of cotransporter FCCP confirm the M^+^/OH^−^ symport mechanism. The selectivity of Rb^+^ over K^+^ (${\text{P}}_{\text{K}}^{+}$/${\text{P}}_{\text{Rb}}^{+}$ = 0.48) indicates a sandwich interaction between the ions and B18C6. Combined with the channel current signal of **LC**, a tetrameric aggregate is proposed as the main active structure of ion transport via a relay mechanism. In addition, the anticancer activity of **LC** on HeLa cells provides a possibility for its application in cancer chemotherapy. Through reasonable molecular design, we hope to provide new opportunities for the development of more effective ion transporters with well-defined structures.

## Data Availability Statement

The original contributions presented in the study are included in the article/supplementary material, further inquiries can be directed to the corresponding author.

## Author Contributions

CB proposed and supervised the project. CW and HY carried out the synthesis, characterizations, and data collection. YX and SP performed the experiments of cytotoxicity assay. CB and LZ oversaw the paper with edits from all authors. All the authors discussed the results and commented on the manuscript.

## Conflict of Interest

The authors declare that the research was conducted in the absence of any commercial or financial relationships that could be construed as a potential conflict of interest.
